# Data from subjects receiving intrathecal laronidase for cervical spinal stenosis due to mucopolysaccharidosis type I

**DOI:** 10.1016/j.dib.2015.08.004

**Published:** 2015-08-20

**Authors:** P.I. Dickson, I. Kaitila, P. Harmatz, A. Mlikotic, A.H. Chen, A. Victoroff, M.B. Passage, J. Madden, S.Q. Le, D.E. Naylor, The Mucopolysaccharidosis I Intrathecal Research Collaborative

**Affiliations:** a Department of Pediatrics, Los Angeles Biomedical Research Institute at Harbor‐UCLA Medical Center, Torrance, CA, USA; b Medical Genetics, University of Helsinki and Helsinki University Hospital, Helsinki, Finland; c UCSF Benioff Children׳s Hospital Oakland, Oakland, CA, USA; d Department of Radiology, Los Angeles Biomedical Research Institute at Harbor-UCLA Medical Center, Torrance, CA, USA; e Department of Neurology, Los Angeles Biomedical Research Institute at Harbor‐UCLA Medical Center, Torrance, CA, USA

## Abstract

Five subjects with mucopolysaccharidosis type I and symptomatic cervical spinal stenosis received intrathecal laronidase in a 4-month pilot study and/or a 12-month extension study [1]. Clinical descriptions of study subjects, nonserious adverse events, individual data tables, and scoring system methods are provided. There were ten nonserious adverse events that occurred in more than one study subject. Somatosensory evoked potentials were absent in two subjects and normal in two subjects, limiting their utility as an endpoint. There were no significant changes in magnetic resonance imaging of cervical spinal cord or brain, pulmonary function tests, or cerebrospinal fluid opening pressure. These data are presented along with the scoring methods used in evaluation of the study subjects.

## **Specifications** table

**Table:** 

Subject area	*Biology*
More specific subject area	*Inborn errors of metabolism*
Type of data	*Text files, tables, figures*
How data was acquired	*Clinical evaluations, magnetic resonance imaging, nerve conduction studies, spirography, lumbar spinal puncture*
Data format	*Raw, processed*
Experimental factors	*Subjects were treated with intrathecal laronidase, and pre- and post-treatment data are presented*
Experimental features	*Clinical data are presented descriptively. Adverse events were tabulated based on subject reporting. Magnetic resonance imaging data were scored for spinal cord compression, hydrocephalus, white matter hyperintensities, and cystic or cribriform changes. Pulmonary function tests, cerebrospinal fluid opening pressure and somatosensory evoked potentials were obtained in standard clinical fashion.*
Data source location	*N/A (multicenter clinical trial)*
Data accessibility	*N/A*

## Value of the data

• There are very few clinical data reports of cervical spinal stenosis due to mucopolysaccharidosis type I, or attempts at treatment.
• Quantitative as well as qualitative data are presented.
• The data may inform future studies of intrathecal enzyme replacement therapy for lysosomal storage disorders.

## 1 Experimental design, materials and methods

### 1.1 Subjects

Study subjects are narratively described in data file 1. They were treated with intrathecal laronidase as described in Ref. [1].

### 1.2 Measures of safety

To evaluate possible adverse effects of study treatments, participants had physical and neurologic examination before and after each study treatment. All new physical complaints were evaluated and recorded including their severity and attribution to study treatments. Table 1 lists nonserious adverse events.

### 1.3 Objective measures of efficacy

**Fig. 1:**
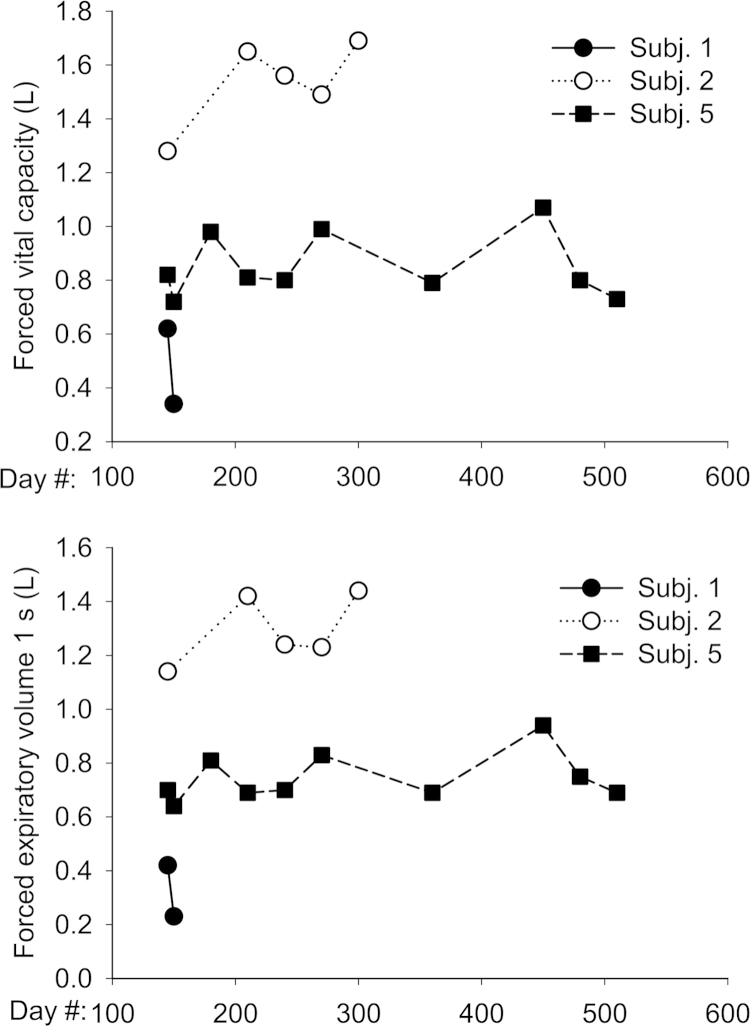
Pulmonary function tests. Subjects in the extension study underwent pulmonary function tests by spirometry at each visit.

**Fig. 2:**
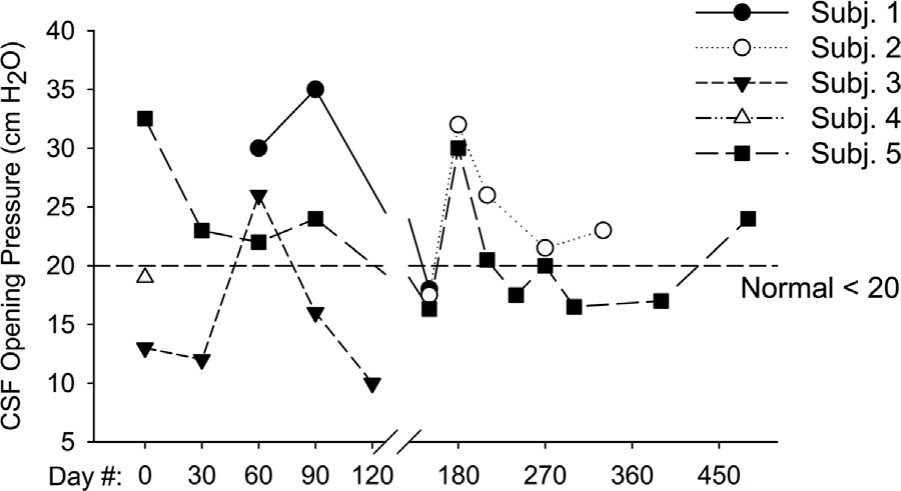
CSF opening pressure. We measured opening pressure prior to each dose of intrathecal laronidase by manometry at the level of the right atrium. We did not obtain opening pressure measurements at Days 0 and 30 for subject 1 due to technical issues performing the lumbar puncture during those visits. Subjects 2–4 had a history of hydrocephalus and implanted CSF drainage shunts.

Response to treatment was assessed using a combination of subjective and objective measures. We evaluated somatosensory evoked potentials in the upper and lower extremity as per [2]. Results are shown in Table 2. MRI of brain and spinal cord were obtained to assess degree of cord compression and measurement of meningeal thickness was taken. MRI were performed using a 1.5-Tesla GE LX9.1. Brain imaging included sagittal T1-weighted, axial FLAIR, axial T2-weighted and axial diffusion-weighted images. Sagittal T1- and T2-weighted images of the whole spine and axial T1-weighted images of the cervical spine were obtained. Axial T1-weighted studies of the cervical spine were used to score spinal cord compression according to the methods of Houten and Cooper [3]. Brain images were evaluated for abnormal signal intensity in T2, enlargement of perivascular spaces, and ventricular size as per Matheus et al. [4]. Results are shown in Tables 3–7. Subjects enrolled in the extension study also underwent pulmonary function testing using spirometry (Fig. 1). Cerebrospinal fluid glycosaminoglycans were measured at Seattle Children׳s Hospital using a clinically-available test (Fig. 2). The laboratory uses a dimethylene blue dye-binding assay to quantitate total glycosaminoglycans [5]. Functional Independence Measure (FIM) score and Japanese Orthopedic Association (JOA) score measures were used to assess any changes in functional status and myelopathy. Results are shown in Fig. 1 of Ref. [1]. Scoring criteria for JOA and FIM are given in data files 2 and 3. The grading systems that were used to indicate the severity of spinal cord compression and brain imaging findings are given in data file 4.

## Figures and Tables

**Table 1:** Nonserious adverse events.

**Events occurring in >1 study subject**	**Events occurring in 1 study subject**
Headache	Anemia
Neck pain	Nausea
Hypoxemia	Pneumonia
Desaturation during procedure	Tingling
Elevated CSF opening pressure	Decreased sensation right hand
Back pain	Blurred vision
Myoclonic-like twitches	Decreased visual acuity
Diarrhea/gastroenteritis	Weakness
Upper respiratory infection	Insomnia
Fever	Elevated serum phosphorus
	Decreased serum protein
	Decreased serum albumin
	Elevated CSF protein
	Elevated CSF leukocyte count
	Hip and thigh pain
	Pruritus (with fentanyl)
	Pneumocephaly
	Dizziness
	Gradual turning outward of right foot hampering walking and balance
	Abdominal pain
	Clumsiness in leg
	Dyspnea and cough
	Joint Pain
	Hand cramps
	Perineal muscle cramps
	Tachycardia at rest
	Sore throat
	“Shaking while laying down”/“tremor”
	Abdominal cramping / dysmenorrhea
	Facial flushing
	Urinary urgency
	Influenza
	Leg weakness
	Urticaria
	Melena
	Malaise

CSF: cerebrospinal fluid.

**Table 2:** Somatosensory evoked potentials. Intrasubject change (absolute) in latency (ms) during the pilot and extension studies for subjects with both baseline and end-study measurements.

**4-Month pilot study**				**12-Month extension study**
Subject number:	1	3	5	5
Median nerve				
N9-N13A	Absent	0.80	Absent	Absent
N9-N13B	Absent^a^	−0.60	Absent	Absent
N9-P13	Absent	1.10	Absent	Absent
N13A-N20	Absent	−0.40	Absent^a^	6.5

Posterior tibial nerve				
N22-P30	Absent	1.00	Absent	Absent
N22-P40	Absent	0.80	2.40	8.8

a N9–N13B was present at baseline in subject 1 at a latency of 3.30 ms but absent at the Day 120 visit. N13A-N20 was present in subject 5 at the Day 120 visit at a latency of 10.8 ms but absent at baseline. Subject 2 did not have baseline measurements (the subject was not enrolled in the pilot study). Subject 4 did not have day 120 measurements (termination of participation due to subject death).

**Table 3:** MRI cervical spinal cord compression (maximum grade).

**4-Month pilot study**			**12-Month extension study**		
Subject number	Baseline	End of study	Baseline	Six months	End of study
1	3	3	3	N/A	N/A
2	N/A	N/A	3	3	N/A
3	2 to 3	2 to 3	N/A	N/A	N/A
4	2	N/A	N/A	N/A	N/A
5	1	ND	1	2	2

Grading system: 0, 360° cushion of CSF around cord; 1, loss of CSF cushion w/o indentation of the cord but may have slight anterior cord flattening; 2, mild spinal cord compression; and 3, severe cord compression.

**Table 4:** Brain MRI abnormal T2 signal intensity.

**4-Month pilot study**			**12-Month extension study**		
Subject number	Baseline	End of Study	Baseline	Six months	End of study
1	2	2	2	N/A	N/A
2	N/A	N/A	0	0	N/A
3	0	0	N/A	N/A	N/A
4	2	N/A	N/A	N/A	N/A
5	1	1	1	2	2

Grading system: score signal changes on T2-weighted images as 0, absent; 1, patchy and confined to the periventricular area; 2, patchy but in other white matter areas as well as periventricular; and 3, diffuse.

**Table 5:** Brain MRI enlargement of perivascular spaces.

**4-Month pilot study**			**12-Month extension study**		
Subject number	Baseline	End of study	Baseline	Six months	End of study
1	2	2	3	N/A	N/A
2	N/A	N/A	1	1	N/A
3	0	0	N/A	N/A	N/A
4	2	N/A	N/A	N/A	N/A
5	3	2	3	2	2

Grading system: 0, no enlargement; 1, <3 mm enlargement; 2, between 3 and 8 mm enlargement; and 3, >8 mm enlargement.

**Table 6:** Brain MRI frontal-occipital horn ratio.

**4-Month pilot study**			**12-Month extension study**		
Subject number	Baseline	End of study	Baseline	Six months	End of study
1	0.418	0.406	0.368	N/A	N/A
2	N/A	N/A	0.472	0.472	N/A
3	0.767	0.752	N/A	N/A	N/A
4	0.547	N/A	N/A	N/A	N/A
5	0.385	0.385	0.385	0.588	0.598

FOR is calculated as follows: measure a ratio between the frontal and occipital horns of the lateral ventricle using the maximum distance between the outer borders of the frontal horns added to the distance between the outer borders of occipital horns, then divide by twice the maximum biparietal diameter.

**Table 7:** Brain MRI atrophy and megacisterna magna.

**4-Month pilot study**			**12-Month extension study**		
Subject number	Baseline	End of study	Baseline	Six months	End of study
Brain atrophy					
1	1	1	1	N/A	N/A
2	N/A	N/A	0	0	N/A
3	0	0	N/A	N/A	N/A
4	0	N/A	N/A	N/A	N/A
5	1	1	1	1	1

Megacisterna magna					
1	1	1	1	N/A	N/A
2	N/A	N/A	1	1	N/A
3	1	1	N/A	N/A	N/A
4	0	N/A	N/A	N/A	N/A
5	0	0	0	0	0

Grading system: 0, absent; 1, mild; 2, moderate; and 3, severe.
