# Supplementary material for: Data from subjects receiving intrathecal laronidase for cervical spinal stenosis due to mucopolysaccharidosis type I
Source: Data Brief. 2015 Aug 20;5:71–6. doi: 10.1016/j.dib.2015.08.004 (PMC4573094; doi:10.1016/j.dib.2015.08.004)
Supplement: Supplementary file 1 — Supplementary data [file mmc1.doc]

Data file 1. Clinical descriptions of the study subjects.

**Study Subject 1**

The study subject, a woman in her thirties with the Hurler-Scheie form of MPS I, was screened at baseline in November 2005. MPS I status was diagnosed at age 2 ½ years and verified by alpha-l-iduronidase deficiency on leukocyte enzyme assay. *IDUA* mutation analysis was not performed. Treatment with intravenous laronidase began at age 29 years. She had multiple joint contractures which restrict range of motion. She had valvular heart disease, with pedal edema, and restrictive lung disease. At the time of enrollment, her FEV-1 was 25% of predicted, and she could not complete a sentence in one breath. At that time, she did not use supplemental oxygen. She was blind in her right eye, sensing only light, and had myopia and retinal detachment in her left eye. She also had glaucoma. She had severe scoliosis, with a 43% rotatory dextro convex curvature. She also had cervical and thoracic spinal stenosis and thoracic spinal disc herniation at multiple levels. She had many cord compression symptoms, including pain in the lower extremities, inability to ambulate or stand unassisted, reduced strength, bladder and bowel incontinence. These were long-standing and present for years (at least 2 years) in all cases. She was wheelchair-bound and could not transfer unassisted. She required assistance for all activities of daily living except feeding. Her neurologic examination showed sensory and motor deficits of the lower extremities with increased lower extremity tone and increased reflexes. Spinal MRI showed cervical and thoracic spinal cord compression. Somatosensory evoked potentials were abnormal and consistent with spinal cord compression. She had been evaluated by a neurosurgeon, and spinal decompression surgery was not recommended.

**Study Subject 2**

Subject 2 was a teenage girl with the Hurler-Scheie form of MPS I diagnosed at age 16 months by enzyme assay and molecular genetics revealing compound heterozygosity for W402X and L238Q in the *IDUA* gene. Clinical features of MPS I included coarse facial features, restricted joint motion, dysostosis multiplex, corneal clouding, reduced airway and respiratory function (requiring intermittent bronchodilator treatment but no daily treatment and no oxygen required), valvular heart disease (mitral stenosis and mitral valve prolapse), reduced growth rate, and umbilical hernia. Neurological features include spinal cord compression and hydrocephalus. Hydrocephalus was diagnosed at age 7 years, and a ventriculoperitoneal shunt was placed at that time. She suffered from frequent migraine headaches, requiring daily amitryptiline and intermittent non-steroidal anti-inflammatory drugs and anti-emetics. The headaches occured almost daily in the bitemporal-frontal region, and have prompted visits to the emergency room approximately monthly. Spinal cord compression was diagnosed at age 13 years by MRI. Symptoms included pain in her legs at night beginning at age 11 years, numbness and tingling in her feet at least once per month since age 12 years, reduced strength in her right hand, resulting in “sloppy handwriting” and difficulty cutting her meat since age 12 years, and fatigue with long walks (around 2 blocks) beginning approximately 6 months prior to enrolment. She did not have bowel or bladder incontinence. Her baseline CSF evaluation showed elevated spinal fluid protein at 604 mg/dL, felt to be due to reduced CSF flow around the site of compression. The study subject had been undergoing treatment off-study beginning at age 13 years with IT laronidase at 1-2 month intervals for six doses between August 2006 and February 2007, followed by a maintenance schedule of every 3 months until October 2, 2007. She was screened and enrolled on January 22, 2008.

**Study Subject 3**

Study subject 3 was a woman with Scheie in her twenties. *IDUA* mutations were W402X/L526P. Treatment with intravenous laronidase began in 2005 at age 22 years. Screening for the study was performed on June 18, 2007, at age 24 years. She had a history of valvular heart disease (aortic and mitral stenosis) and an umbilical hernia since childhood. She had developed joint pains, carpal tunnel syndrome, restrictive lung disease, splenomegaly and coarse features. Spinal cord compression was diagnosed in March 2007, after she presented with numbness and tingling of the hands and feet, nighttime leg pain, upper back pain, and intermittent urinary incontinence. An MRI of the cervical spine showed canal stenosis at C2-C4 without cord flattening or signal change. Other medical problems included hydrocephalus requiring a lumbo-peritoneal shunt, corneal clouding, hyperopia, restrictive lung disease, tachycardia requiring an implanted cardiac monitor (subsequently removed), hypothyroidism, restless legs, and headaches.

**Study Subject 4**

Subject 4 was a male with MPS I in his teens who was screened for enrolment in January 2008. *IDUA* gene mutations were W402X/L238Q. Spinal cord compression had developed in 2006, with intermittent numbness and tingling of the hands and feet, and leg pain. There was no bladder or bowel incontinence, and no loss of strength or mobility. Neurologic examination showed subtle reflex asymmetries, contractures, short digit span, poor serial 7’s, and possible upgoing right toe. MRI of the spine showed spinal cord compression at C1-C3 and in the lumbar region. Somatosensory evoked potentials were abnormal in the lower extremity. Other medical problems included hydrocephalus, which had been treated with a ventriculoperitoneal shunt, seasonal (mild intermittent) asthma, umbilical hernia, carpal tunnel syndrome, tonsillectomy and adenoidectomy, mild mitral valve regurgitation and questionable slight mitral valve prolapse, astigmatism, and eczema. Treatment with intravenous laronidase began at age 9 years, and though occasional hives were noted, there were no systemic allergic reactions. His primary medical doctor in charge of infusions confirmed that this was the case.

**Study Subject 5**

Subject 5 was a woman with Hurler-Scheie syndrome in her twenties who began the study June 14, 2007 at the study site in Helsinki. At the age of 3 years and 6 months the patient was referred for progressive joint stiffness in the hands. The physical findings showed generalized stiffness of joints in the upper extremities, thickened fingers, mild hirsutism, III/VI systolic heart murmur, tiny umbilical hernia, and mild diffuse corneal clouding. Skeletal radiographs showed dysostotic abnormalities of the phalanges in her hands. Urinary excretion of glycosaminoglycans was increased, and alpha-l-iduronidase activity of cultured skin fibroblasts was negligible, 1.1 nmol/h/mg (normal range 200-500). Molecular analysis of the *IDUA* gene demonstrated Q70X/396insAC indicating a compound heterozygosity. The intellectual developmental history in contrast to her progressive physical handicap had been normal. The patient had developed the characteristic somatic problems of the lysosomal storage disease. There had been a progressive limitation of the joint movements in the upper extremities, and from the age of about 8 years, in the lower extremities. She experienced physical limitations in walking, moving around and in every-day tasks including dressing and grooming. Growth in height had been normal up to 7-8 years of age after which severe growth retardation had ensued with a final adult height of 135 cm. There was corneal clouding and an umbilical hernia requiring repair. Pubertal development had been delayed, and menarche had ensued at the age of 17 years. She experienced dyspnea on exertion and orthopnea, requiring her to sleep in an upright position. Pulmonary function studies had shown that there were both restrictive pulmonary function and obstructive sleep apnea. Echocardiography showed thickened and deformed mitral and aortic valves and minor valve insufficiencies, but no right-sided strain. At the age of 15 years cramps, numbness and muscular weakness of the extremities had developed suggesting tetraparesis, and cervical spinal stenosis of C1 to C5 was confirmed by somatosensory evoked potentials and cervical MRI. At the age of 16 years she underwent laminectomy of the C1-C3, and the symptoms and signs of tetraparesis resolved. Treatment with intravenous enzyme replacement therapy began at age 22 years. In 2004, gradual spasticity of the lower legs developed and the subject was diagnosed with recurrence of cervical spinal stenosis. The risks of an operative intervention most likely exceed the possible benefits, and thus the patient was considered for a trial of intrathecal enzyme replacement therapy.
