# Supplementary material for: Data from subjects receiving intrathecal laronidase for cervical spinal stenosis due to mucopolysaccharidosis type I
Source: Data Brief. 2015 Aug 20;5:71–6. doi: 10.1016/j.dib.2015.08.004 (PMC4573094; doi:10.1016/j.dib.2015.08.004)
Supplement: Supplementary file 2 — Supplementary data [file mmc2.doc]

Data file 2. Revised Japanese Orthopedic Association (JOA) scale

**Motor function**

Fingers

0 – Unable to feed oneself

1 – Unable to use knife and fork but can eat with spoon

2 – Able to use knife and fork with much difficulty

3 – Able to use knife and fork with slight difficulty

4 – Normal

Shoulder and elbow – evaluated by manual muscle test (MMT)^[[1]](#footnote-1)^ of deltoid or biceps muscles, whichever is weaker.

-2 – MMT 2 or less

-1 – MMT 2.5 – 3.5

-0.5 – MMT 4 – 4.5

0 – MMT 5

Lower extremity

0 – Unable to stand up and walk by any means

0.5 – Able to stand up but not walk

1 – Unable to walk without a cane or other support on a level surface

1.5 – Able to walk without support but with a clumsy gait

2 – Walks independently on a level surface but needs support on stairs

2.5 – Able to walk independently upstairs but needs support downstairs

3 – Capable of fast but clumsy walking

4 – Normal

**Sensory function**

Upper extremity

0 – Complete loss of touch and pain sensation

0.5 – 50% or less normal sensation and/or severe pain or numbness

1 – More than 60% normal sensation and/or moderate pain or numbness

1.5 – Subjective numbness of slight degree without any objective sensory deficit

2 – Normal

Trunk

0 – Complete loss of touch and pain sensation

0.5 – 50% or less normal sensation and/or severe pain or numbness

1 – More than 60% normal sensation and/or moderate pain or numbness

1.5 – Subjective numbness of slight degree without any objective sensory deficit

2 – Normal

Lower extremity

0 – Complete loss of touch and pain sensation

0.5 – 50% or less normal sensation and/or severe pain or numbness

1 – More than 60% normal sensation and/or moderate pain or numbness

1.5 – Subjective numbness of slight degree without any objective sensory deficit

2 – Normal

Bladder function

0 – Urinary retention and/or incontinence

1 – Sensation of retention and/or dribbling and/or thin stream and/or incomplete continence

2 – Urinary retardation and/or pollakiuria

3 – Normal

A total score between -2 and 17 was recorded for each subject at each evaluation. Subscores for motor and sensation and a lower extremity subscore were calculated.

1. Manual muscle test scoring system:

   0 = total paralysis

   1 = palpable or visible contraction

   2 = active movement, full range of motion with gravity eliminated

   2.5

   3 = active movement, full range of motion against gravity

   3.5

   4 = active movement, full range of motion against moderate resistance

   4.5

   5 = active movement, full range of motion against full resistance (normal)

   NT == not testable [↑](#footnote-ref-1)
