# Supplementary material for: Data from subjects receiving intrathecal laronidase for cervical spinal stenosis due to mucopolysaccharidosis type I
Source: Data Brief. 2015 Aug 20;5:71–6. doi: 10.1016/j.dib.2015.08.004 (PMC4573094; doi:10.1016/j.dib.2015.08.004)
Supplement: Supplementary file 3 — Supplementary data [file mmc3.doc]

Data file 3: Functional Independence Measure (FIM) score

Each area of functioning was evaluated and scored by the subject on a 7 point scale. Scores of 6 or 7 were considered “Independent,” and 5 or below considered “Dependent.”

7 = Complete independence. The activity is typically performed safely, without modification, assistive devices or aids, and within reasonable time

6 = Modified independence. The activity requires an assistive device and/or more than reasonable time and/or is not performed safely.

5 = Supervision or setup. No physical assistance is needed, but cuing, coaxing, or setup is required.

4 = Minimal contact assistance. Subject requires no more than touching and expends 75% or more of the effort required in the activity.

3 = Moderate assistance. Subject requires more than touching and expends 50-75% of the effort required in the activity.

2 = Maximal assistance. Subject expends 25-50% of the effort required in the activity.

1 = Total assistance. Subject expends 0-25% of the effort required in the activity.

Areas of functioning addressed:

Self-care -- Eating, grooming, bathing, dressing-upper body, dressing-lower body, and toileting were evaluated on the 7-point scale

Sphincter control -- Bladder and bowel management were each rated on the 7-point scale.

Mobility -- Transfer to bed, chair, or wheelchair, transfer to toilet, and transfer to tub or shower were graded on the scale.

Locomotion -- Walking/wheelchair and stairs

A total FIM score was assigned. A score of 1 was entered if the subject was not testable in that area. The score was an integer from 13 to 91.
