# Supplementary material for: Data from subjects receiving intrathecal laronidase for cervical spinal stenosis due to mucopolysaccharidosis type I
Source: Data Brief. 2015 Aug 20;5:71–6. doi: 10.1016/j.dib.2015.08.004 (PMC4573094; doi:10.1016/j.dib.2015.08.004)
Supplement: Supplementary file 4 — Supplementary data [file mmc4.doc]

Data file 4. MRI of brain and spine: scoring system

**Spinal cord compression**

Grade 0: 360-degree cushion of cerebrospinal fluid around the spinal cord.

Grade 1: loss of cerebrospinal fluid cushion without indentation of the spinal
cord. May have slight anterior cord flattening.

Grade 2: mild spinal cord compression.

Grade 3: severe spinal cord compression

**Abnormal signal intensity in brain**

Lesions were estimated as to size (3 dimensions) and number, and were divided by location into basal ganglia, white matter, and cortex. Signal changes observed on T2 images were graded on a scale from 0-3, where 0 is absent, 1 is patchy and confined to the periventricular area, 2 is patchy but in other white matter areas as well as periventricular, and 3 is diffuse.

**Enlargement of perivascular spaces in brain**

Enlargement of the perivascular space were classified in five brain regions: periventricular and subcortical white matter, corpus callosum, basal ganglia, thalami, and brainstem. These regions were scored for enlargement on a scale from 0-3, where 0 is no enlargement, 1 is < 3mm enlargement, 2 is between 3 and 8 mm enlargement, and 3 is > 8 mm of enlargement.

**Ventricular size**

Ventricular size or hydrocephalus were graded using a semi-quantitative frontal occipital horn ratio (FOR). A ratio between the frontal and occipital horns of the lateral ventricle was measured using the maximum distance between the outer borders of the frontal horns added to the distance between the outer borders of occipital horns, divided by twice the maximum biparietal diameter.

Other findings, such as brain atrophy and megacisterna magna were graded on a scale from 0-3, with 0 as absent, 1 as mild, 2 as moderate, and 3 as severe. Additional abnormalities were also noted.
